# A Human-Derived Putative Probiotic, *Ligilactobacillus salivarius* MCC0226 with Adhesion, Antipathogenic, and Epithelial Gene-Modulatory Properties

**DOI:** 10.3390/foods15203649

**Published:** 2026-10-14

**Authors:** Saily Harkal, Neelam Kapse, Deepa Shetty, Vaishali Javadekar-Subhedar, Shilpa Wagh, Sumit S. Dagar, Prashant K. Dhakephalkar

**Affiliations:** 1 Bioenergy Group, MACS- Agharkar Research Institute, G.G. Agarkar Road, Pune 411004, Maharashtra, India; sailyharkal@aripune.org (S.H.); deepashetty@aripune.org (D.S.); ssdagar@aripune.org (S.S.D.); 2 Department of Microbiology, Savitribai Phule Pune University, Ganeshkhind Rd, Aundh, Pune 411007, Maharashtra, India; 3 Hi Tech BioSciences India Ltd., Research & Development Center, Plot No. 6 and 8, Ambadvet Industrial Estate, PO Paud, Pune 412108, Maharashtra, India

**Keywords:** *Ligilactobacillus salivarius*, intestinal colonization, tight-junction proteins, whole-genome sequencing, functional probiotic

## Abstract

*Ligilactobacillus salivarius* is a well-recognized probiotic species; however, the mechanisms by which individual strains contribute to gut ecosystem homeostasis and protection of the intestinal epithelial barrier remain poorly understood. This study comprehensively characterized a human-derived *L. salivarius* MCC0226 using an integrated genomic and functional approach to evaluate its probiotic potential. Whole-genome sequencing identified genes associated with acid and bile tolerance, adhesion, exopolysaccharide biosynthesis, oxidative stress response, bacteriocin production, bile salt hydrolase activity, and amino acid biosynthesis. At the same time, genomic safety analysis confirmed the absence of virulence factors and clinically relevant transferable antimicrobial resistance determinants. MCC0226 exhibited high survival under simulated gastric and intestinal conditions, produced 0.64 g L^−1^ exopolysaccharides, and showed strong adhesion to HT-29 and Caco-2 intestinal epithelial cells. The strain demonstrated broad-spectrum antimicrobial activity against 13 clinically relevant pathogens, reduced adhesion of *Salmonella* Typhimurium and *Clostridioides difficile* on HT-29 cells, co-aggregated with enteric pathogens, and produced hydrogen peroxide. Functional metabolite analysis revealed antioxidant activity, cholesterol assimilation under simulated intestinal and colonic conditions, positive bile salt hydrolase activity, and production of lactic, acetic, and butyric acids during inulin fermentation. Furthermore, MCC0226 significantly modulated the expression of epithelial junction-associated genes in HT-29 cells, including OCLN, CLDN3, CDH1, and CLDN2, while ZO-1 remained essentially unaffected. The combined genomic and in vitro evidence indicates that MCC0226 possesses multiple complementary mechanisms supporting intestinal colonization, microbial modulation, and regulation of epithelial junction-associated gene expression, highlighting its potential as a multifunctional probiotic candidate for functional food and nutraceutical applications.

## 1. Introduction

The human gastrointestinal tract harbours a complex and dynamic microbial ecosystem that plays a central role in digestion, nutrient metabolism, immune development, and maintenance of intestinal homeostasis. The intestinal epithelium forms a selective barrier between the luminal environment and the host, allowing nutrient absorption while restricting the passage of pathogens, toxins, and inflammatory molecules. This barrier is maintained by an integrated network of epithelial cells, mucus, commensal microorganisms, microbial metabolites, and intercellular junctional complexes, particularly tight junction proteins. Tight junction proteins, including occludins, claudins, and zonula occludens proteins, play a central role in maintaining epithelial integrity. Disruption of this barrier increases intestinal permeability and contributes to gastrointestinal infections, inflammatory bowel disease, metabolic disorders, and systemic inflammation. Consequently, probiotics capable of strengthening epithelial barrier function and maintaining microbial equilibrium have gained increasing attention as functional food ingredients with disease-preventive potential [1,2,3].

Growing evidence indicates that probiotic-mediated health benefits extend beyond modulation of microbial composition to include the preservation of intestinal epithelial barrier integrity, regulation of host immune responses, and the production of bioactive metabolites that contribute to gut homeostasis [2,3]. Since probiotic effects are highly strain-specific and dose-dependent, rigorous phenotypic and genomic characterization of each candidate strain is essential before its application in foods, feeds, or clinical interventions [4].

Among the lactic acid bacteria (LAB) explored for probiotic applications, *Ligilactobacillus salivarius* (formerly *Lactobacillus salivarius*) has attracted considerable interest as a host-adapted member of the human oropharyngeal and gastrointestinal microbiota with well-documented probiotic and disease-preventive properties [5]. The species has been granted Qualified Presumption of Safety (QPS) status by the European Food Safety Authority and has been isolated from breast milk, feces, the oral cavity, and the vaginal microbiota [3,6]. Numerous *L. salivarius* strains exhibit desirable probiotic characteristics, including resistance to acidic and bile conditions, strong adhesion to intestinal epithelial cells, antioxidant activity, cholesterol-lowering potential, and production of bacteriocins with inhibitory activity against enteric pathogens [3,7]. Recent studies have also suggested that *L. salivarius* contributes to maintaining gut homeostasis by regulating interactions among intestinal microbes and epithelial barrier function [3].

As with other candidate LAB, the probiotic potential of *L. salivarius* strains has traditionally been established through in vitro phenotypic assays assessing gastrointestinal survival, adhesion, antimicrobial activity, and related functional attributes. Strain L33, isolated from the broiler chicken gut has been evaluated for resistance to simulated digestive conditions, antibiotic susceptibility, adherence, and antimicrobial activity [8]; strain F14, isolated from tribal fecal samples has been characterized for acid and bile tolerance, adhesion, and pathogen inhibition [9]; strain CNCM I-4866 has demonstrated the ability to improve intestinal permeability, modulate cytokine production, inhibit pathogens, and adhere efficiently to intestinal epithelial cells [10]. Although these studies establish important probiotic phenotypes, they primarily investigate individual functional properties rather than examining how these traits collectively contribute to the protection of the intestinal ecosystem and epithelial barrier.

Protection of the gut ecosystem is a multifactorial process involving coordinated interactions between probiotic bacteria, intestinal epithelial cells, resident microbiota, and invading pathogens. Adhesion-associated proteins and exopolysaccharides facilitate attachment to mucus and epithelial surfaces, promoting transient colonization and competitive exclusion of pathogens. Antimicrobial metabolites, including organic acids, hydrogen peroxide, and bacteriocins, suppress pathogen growth and limit epithelial colonization, while fermentation-derived short-chain fatty acids contribute to epithelial energy metabolism, mucus production, and regulation of inflammatory responses. Together, these complementary mechanisms enable probiotics to preserve epithelial integrity and maintain intestinal homeostasis [2,3].

Despite the growing body of strain-specific evidence, the molecular mechanisms by which *L. salivarius* preserves intestinal epithelial barrier integrity and contributes to gut ecosystem homeostasis remain unclear, as most studies examine isolated phenotypes rather than an integrated genomic–functional approach. This study addresses these gaps by characterizing *L. salivarius* MCC0226 through an integrated genomic–functional approach that combines whole-genome mining and annotation with targeted phenotypic assays, epithelial cell models, metabolite profiling, and pathogen exclusion experiments. This study aimed to elucidate the mechanistic basis by which *L. salivarius* MCC0226 protects the intestinal ecosystem. We integrated phenotypic characterization, epithelial cell assays, metabolite profiling, pathogen exclusion assays, and whole-genome mining to determine how MCC0226 survives gastrointestinal transit, colonizes intestinal epithelium, remodels microbial interactions, produces functional metabolites, and reinforces epithelial tight junction integrity. By linking metabolic output, pathogen exclusion, and barrier reinforcement to genomic features, this work advances from fragmented phenotypes to a mechanistic, systems-level understanding of MCC0226’s protective role in the intestinal ecosystem.

## 2. Materials and Methods

### 2.1. Bacterial Strain and Culture Conditions

The *L. salivarius* strain was isolated from a fecal sample collected from a healthy 30-year-old adult male volunteer and was cultured anaerobically in De Man, Rogosa, and Sharpe (MRS) medium (Himedia: GM369, Mumbai, India) with L-cysteine HCl (SRL: 69029, Mumbai, India) at 37 °C. *L. salivarius* strain has been deposited at the National Center for Microbial Resource (NCMR) in Pune, India, and has been assigned accession number MCC0226.

### 2.2. Whole Genome Sequencing, Assembly, and Annotation

Whole-genome sequencing of MCC0226 was performed using the Oxford Nanopore sequencing platform (flow cell FLO-MIN106D). Raw sequencing reads were assembled de novo using Flye v2.9 with default parameters. The MCC0226 genome has been deposited in GenBank under the nucleotide sequence accession number JBANDJ000000000.1. Genome annotation was carried out using the RAST server [11], and the annotated genome was manually curated to identify genes associated with probiotic functionality, including stress tolerance, adhesion, exopolysaccharide biosynthesis, oxidative stress response, and bacteriocin production. Bacteriocin biosynthetic gene clusters were predicted using BAGEL 4.0.

Carbohydrate-active enzymes (CAZymes) in MCC0226 were identified using the dbCAN3 meta server based on the CAZy database. The predicted protein sequences from the annotated genome were analyzed using the HMMER module with default parameters. Identified CAZymes were classified into glycoside hydrolase (GH), glycosyltransferase (GT), carbohydrate esterase (CE), carbohydrate-binding module (CBM), and other CAZy families. CAZymes associated with fructan metabolism were manually curated for downstream analysis.

### 2.3. Genotypic and Phenotypic Safety Assessment

Genomic safety was evaluated using the Centre for Genomic Epidemiology (CGE) platform, including CARD-RGI, ResFinder 4.7.2, VirulenceFinder 2.0, and PlasmidFinder 2.1, to identify antimicrobial resistance genes, virulence determinants, toxins, and plasmid-associated sequences. The pathogenic potential of MCC0226 was further assessed using PathogenFinder 1.1.

The antibiotic susceptibility profile of MCC0226 was determined using the E-test method with Ezy MIC™ strips (HiMedia Laboratories, Mumbai, India). E-test strips containing antibiotic concentrations ranging from 0.016 to 256 μg/mL were used. Briefly, 100 μL of MCC0226 (10^8^ cells/mL) was uniformly spread onto MRS agar supplemented with cysteine. The appropriate E-test strip was then placed at the center of each agar plate, followed by incubation at 37 °C for 24 h. The minimum inhibitory concentration (MIC) was determined as the lowest antibiotic concentration at which no visible bacterial growth was observed. The susceptibility profile of MCC0226 was interpreted according to the applicable microbiological cut-off values established by EFSA, and the strain was categorized as susceptible or resistant accordingly.

### 2.4. Functional Characterization of MCC0226

#### 2.4.1. Assessment of Resistance to Simulated Gastrointestinal Conditions

The gastrointestinal tolerance of MCC0226 was evaluated using simulated gastric and intestinal fluids under strict anaerobic conditions. An overnight culture was harvested after 24 h, washed with sterile MRS medium, and adjusted to a final concentration of 1 × 10^8^ CFU mL^−1^. Gastric tolerance was determined following the method of Ghelardi et al. [12] with minor modifications. In total, 10^8^ cells of MCC0226 were suspended in anaerobically prepared simulated gastric fluid (SGF) containing 0.03 M sodium chloride (SRL: 41721, Mumbai, India), 0.084 M hydrochloric acid (SDFCL: 20125 L25, Mumbai, India), and 0.32% (*w*/*v*) pepsin (HiMedia: GRM084, Mumbai, India) (pH 2.5) and incubated at 37 °C for 2 h. Intestinal tolerance was assessed according to Tokatlı et al. [13] with minor modifications. Cells were exposed to anaerobically prepared simulated intestinal fluid (SIF) comprising 0.3% (*w*/*v*) ox-gall bile salts (DIFCO: 212820, Mumbai, India) and 0.1% (*w*/*v*) pancreatin (HiMedia: RM7384, Mumbai, India) in 0.85% (*w*/*v*) saline (pH 8.0) and incubated at 37 °C for 6 h. At predetermined time intervals, aliquots (100 µL) were serially diluted and plated on MRS agar supplemented with cysteine-HCl. Plates were incubated at 37 °C in an anaerobic glove box, and viable counts were expressed as CFU mL^−1^. All gastrointestinal survival assays were performed under identical anaerobic conditions at 37 °C.

#### 2.4.2. Adhesion Potential

##### Evaluation of the Cell-Surface Properties of Strain MCC0226

Surface-related adhesion properties of MCC0226 were examined through autoaggregation and bacterial adherence to hydrocarbons (BATH) assays. For both assays, a 24-h culture of MCC0226 was washed and resuspended in PBS (Phosphate-Buffered Saline, pH 7.4) for autoaggregation and PUM (Phosphate Urea Magnesium Sulfate, pH 7.1) for hydrophobicity to a final OD_600_ of 1.0.

Autoaggregation was determined by measuring the sedimentation of bacterial suspensions over time according to the protocol of Han et al. [14]. The suspension was incubated under static conditions at 37 °C, and the optical density of the upper layer was recorded at 0, 1, and 4 h. Autoaggregation percentage was calculated as:

% Auto-aggregation = (1 −A_t_/A_0_) × 100

Cell surface hydrophobicity was assessed using the bacterial adherence to hydrocarbons (BATH) assay, as described by Rokana et al. [15]. A 3 mL bacterial suspension was mixed with 1 mL of each hydrocarbon (chloroform, toluene, ethyl acetate, xylene, n-hexane, or hexadecane) in separate tubes. The suspension was incubated at 37 °C for 10 min, vortexed for 15 s, and left undisturbed for 30 min to allow phase separation. After phase separation, the absorbance of the aqueous layer was measured. Hydrophobicity was calculated as the percentage reduction in optical density of the aqueous phase relative to the initial value:

% Hydrophobicity = (OD_i_ − OD_t_/OD_i_) × 100

##### Exopolysaccharide (EPS) Production and Quantification

Exopolysaccharide (EPS) production by MCC0226 was quantified as released extracellular EPS following the protocol of Midik et al. [16] with minor modifications, using trichloroacetic acid (TCA) for protein removal followed by ethanol precipitation of EPS. Briefly, 1% (*v*/*v*) of an overnight culture was inoculated into 10 mL of MRS broth supplemented with cysteine-HCl and incubated at 37 °C for 24 h. Following incubation, 1 mL of 80% (*v*/*v*) TCA was added to the culture, mixed gently, and incubated at −20 °C for 10 min to precipitate proteins. The mixture was centrifuged at 3354× *g* for 20 min at 4 °C; bacterial cells and precipitated proteins were removed as a pellet, while the clarified supernatant containing soluble extracellular EPS was carefully collected. EPS was subsequently precipitated from the clarified supernatant using two volumes of chilled 95% ethanol, incubated at −20 °C for 30 min, recovered by centrifugation, washed with cold ethanol, air-dried, and dissolved in Milli-Q water. The total EPS content was quantified using the phenol–sulphuric acid method [17], with glucose as the standard. EPS concentration was expressed as mg L^−1^.

##### Adhesion to Intestinal Epithelium: HT-29 and Caco-2

The adhesion capacity of MCC0226 to the human intestinal cell lines HT-29 and Caco-2 was determined as described by Sharma et al. [18]. HT-29 (ATCC no. HTB-38) and Caco-2 (ATCC no. HTB-37) cell lines, obtained from the National Centre for Cell Science (NCCS), Pune, India, were maintained in Dulbecco’s Modified Eagle Medium (DMEM; Gibco: 11965092, Waltham, MA, USA). Cells were seeded at a density of 1 × 10^5^ cells/mL onto sterile coverslips placed in 24-well tissue culture plates and incubated until confluent monolayers were obtained. Subsequently, 1 × 10^8^ CFU/mL of MCC0226 was added to each well and incubated at 37 °C for 2 h for HT-29 cells and 1.5 h for Caco-2 cells. After incubation, non-adherent bacteria were removed by gentle washing twice with phosphate-buffered saline (PBS), and the monolayers were fixed with 2% glutaraldehyde overnight at 4 °C. Wells containing only HT-29 or Caco-2 cells served as negative controls. All experiments were performed in triplicate. Bacterial adhesion was assessed by scanning electron microscopy (SEM) as described by Inturri et al. [19]. For quantitative analysis, adherent bacteria were counted directly from SEM micrographs in 25 random fields, the number of bacteria associated with 100 HT-29/Caco-2 cells was recorded, and the results were expressed as the mean number of bacteria adhered per 100 HT-29/Caco-2 cells.

#### 2.4.3. Antagonistic Effects of MCC0226

##### Coaggregation with Pathogens

The coaggregation capacity of MCC0226 against the pathogen strains *Staphylococcus aureus* ATCC 6538, *Enterococcus faecalis* ATCC 29212, *Escherichia coli* ATCC 8739, *Enterobacter aerogenes* ATCC 13048, *Salmonella* Typhimurium ATCC 13311, *Shigella dysenteriae* ATCC 13313, *Klebsiella pneumoniae* ATCC 13883, and *Pseudomonas aeruginosa* ATCC 10145 was evaluated according to the protocol described by Collado et al. [20]. In brief, equal volumes of MCC0226 and each pathogen suspension were mixed (1:1 ratio; final volume 1 mL; initial OD_600_ ≈ 1.0 for both culture and pathogen). The mixtures were placed in sterile tubes, thoroughly vortexed, and incubated under static conditions at 37 °C. Optical density of the upper phase was recorded after 0 h, 1 h, and 4 h (OD_mix_). All assays were performed in triplicate. Percentage coaggregation was calculated using the equation:

%Co-aggregation = [(OD_path_ + OD_cul_)−OD_mix_/(OD_path_ + OD_cul_)] ×100

##### Antimicrobial Activity

Antimicrobial activity of MCC0226 was evaluated by the agar-spot assay and agar-well diffusion assay using cell-free supernatant (CFS).

For the agar-spot assay, 5 µL of MCC0226 (10^8^ cells/mL) was spotted on MRS agar containing cysteine HCl and incubated anaerobically at 37 °C for 24 h [21]. In total, 10^6^ cells/mL of indicator pathogens (*E. coli* ATCC 8739, *S. aureus* ATCC 6538, *E. faecalis* ATCC 29212, *S. dysenteriae* ATCC 13313, *K. pneumoniae* ATCC 13883, *P. aeruginosa* ATCC 10145, *E. aerogenes* ATCC 13048, EHEC O157:H7 ATCC 43888, *S.* Typhimurium ATCC 13311, *Bacillus subtilis* ATCC 6051, *Clostridium perfringens* ATCC 13124, *Bacillus cereus* ATCC 10876, *Yersinia enterocolitica* ATCC 23715, *Clostridioides difficile* ATCC 9689 and *Campylobacter jejuni* ATCC 33560) were overlaid over the probiotic spots, and plates w ere incubated at 37 °C for 24 h.

For the agar well-diffusion method, CFS was prepared by growing MCC0226 in MRS + cysteine broth (37 °C, 24 h), centrifuging (5700× *g*, 10 min), and filter-sterilizing the supernatant (0.22 µm). CFS was concentrated to 50% and 80% using Concentrator Plus. Aliquots of 80% CFS were neutralized (pH 7.0, 4 M NaOH) or treated with proteinase K (1 mg/mL, 37 °C, 2 h; heat-inactivated at 95 °C, 1 min). Pathogen suspensions (0.1 mL, 10^6^ cells/mL) were spread on BHI agar plates. Wells (10 mm) were filled with 100 µL of native, 50%, 80%, neutralized, or proteinase-K-treated CFS, pre-diffused at 4 °C for 2 h, and incubated at 37 °C for 24–48 h. After incubation, inhibition-zone diameters (mm) were measured. Ethanol (70%) and uninoculated medium served as positive and negative controls, respectively.

##### Pathogen Exclusion Assay

The ability of MCC0226 to inhibit the adhesion of pathogens such as *S*. Typhimurium and *C. difficile* to HT-29 intestinal epithelial cells was evaluated using an exclusion assay modified from Choi et al. [22]. HT-29 monolayers grown on coverslips in 24-well tissue-culture plates were treated with 1 mL of MCC0226 suspension (1 × 10^8^ CFU/mL, prepared in antibiotic-free DMEM) and incubated for 1 h at 37 °C under 5% CO_2_. After incubation, non-adherent MCC0226 cells were removed by washing twice with phosphate-buffered saline (PBS). Subsequently, 1 mL of pathogen suspension (1 × 10^8^ CFU/mL prepared in antibiotic-free DMEM) was added to each well and incubated for a further 1 h under the same conditions. Following incubation, non-adherent pathogenic bacteria were removed by washing twice with PBS. The cells were fixed by adding 1 mL of methanol per well for 10 min at room temperature, then briefly air-dried (approximately 3–4 min). Coverslips were Gram-stained and examined under a 100× oil-immersion objective. Twenty to twenty-five random microscopic fields were imaged after Gram staining, and the number of adhered bacterial cells per HT-29 cell was enumerated. Results were expressed as the number of bacterial cells adhered per 100 HT-29 cells, with adhesion of the pathogen alone set at 100% (control).

##### Determination of H_2_O_2_ Production

Hydrogen peroxide production by MCC0226 was determined using a standardized assay according to Martín et al. [23]. Briefly, 500 µL of MCC0226 culture was inoculated into 9 mL of glucose-supplemented PBS (pH 7.4, 0.5 mM glucose) and incubated at 37 °C for 24 h with shaking at 180 rpm. Cells were removed by centrifugation (12,000× *g*, 10 min, 4 °C). An aliquot of the supernatant (0.5 mL) was mixed with horseradish peroxidase (SRL, Mumbai, India) (0.1 mL, 0.01 mg/mL) and o-dianisidine (LOBA CHEMIE: 03220, Mumbai. India) solution (0.01 mL) and incubated at 26 °C for 10 min. The reaction was stopped by adding 4 N HCl (0.02 mL). A blank was prepared identically using glucose-PBS in place of the supernatant. After 5 min, absorbance was measured at 400 nm.

#### 2.4.4. Determination of the Antioxidant Activity

The free radical scavenging activity of MCC0226 was assessed by DPPH assay. The cells were harvested by centrifugation (8586× *g*, 10 min, 4 °C), washed twice, and resuspended in PBS, which was used as the bacterial suspension. In total, 1.0 mL of the bacterial suspension adjusted to 10^9^ cells/mL was mixed with 1.0 mL of DPPH (HiMedia: RM2798, Mumbai, India)–ethanol (Emcure: 1.00983.0, Pune, India) solution (0.2 mM). The resulting mixture was incubated at 25 °C in the dark for 30 min. PBS and a DPPH–ethanol solution were used as controls, while the sample and ethanol were the blanks. After incubation, absorbance was measured at 517 nm in triplicate [24]. Ascorbic acid (10 µg) was used as a reference standard.

#### 2.4.5. Cholesterol Assimilation Ability and Bile Salt Hydrolases (BSHs)

The cholesterol-lowering potential of MCC0226 was evaluated by determining cholesterol assimilation and bile salt hydrolase (BSH) activity, which represent complementary mechanisms involved in probiotic-mediated cholesterol metabolism.

Cholesterol assimilation was determined according to Tomaro-Duchesneau et al. [25] with minor modifications. MCC0226 was inoculated into anaerobically prepared MRS in simulated colonic fluid (SCF) and simulated intestinal fluid (SIF) supplemented with 100 μg mL^−1^ filter-sterilized water-soluble cholesterol (SIGMA ALDRICH: C1145-16, St. Louis, MO, USA) and incubated at 37 °C for 24 h under anaerobic conditions. Following incubation, cultures were centrifuged, and the residual cholesterol in the supernatant was quantified using the o-phthalaldehyde (HiMedia: RM1143, Mumbai, India) method described by Rudel et al. [26]. Appropriate uninoculated controls were included, and all assays were performed in triplicate.

The amount of cholesterol assimilated was calculated as:

Cholesterol assimilated (μg mL^−1^) = Cholesterol 0 h – Cholesterol 24 h

The percentage cholesterol assimilation was calculated using the following equation:

Cholesterol assimilation (%) = (Cholesterol assimilated/Initial cholesterol concentration) × 100

Qualitative BSH activity was determined using the plate assay described by Ahire et al. [27]. Briefly, 5 μL of an overnight-grown MCC0226 culture was spot-inoculated onto MRS agar supplemented with cysteine-HCl and containing either 0.5% (*w*/*v*) sodium glycocholate (HiMedia: GRM8907, Mumbai, India), 0.5% (*w*/*v*) sodium taurodeoxycholate (HiMedia: TC347, Mumbai, India), or 0.3% (*w*/*v*) bile (HiMedia: RM010, Mumbai, India), along with 0.037% (*w*/*v*) CaCl_2_. Plates were incubated at 37 °C for 5 days under microaerophilic conditions. The formation of opaque precipitation zones around the colonies was considered indicative of positive BSH activity.

#### 2.4.6. Short-Chain Fatty Acid (SCFA) Production

The SCFA profile of strain MCC0226 was determined following the method of Renye et al. [28] with modifications. A glucose-free modified MRS (mMRS) basal medium supplemented with L-cysteine HCl was used for the assay. The medium was individually supplemented with 1% (*w*/*v*) fructooligosaccharides (FOS; HiMedia Laboratories, India; RM9582) or 1% (*w*/*v*) inulin (HiMedia Laboratories, India; GRM103) as the sole carbon source. The FOS preparation used in this study is a short-chain fructooligosaccharide preparation with a reported degree of polymerization (DP) of approximately 3–9, whereas the inulin preparation is a long-chain fructan with a reported DP range of approximately 10–60. Glucose-free mMRS without an added carbohydrate served as the negative control, while mMRS supplemented with 1% (*w*/*v*) glucose (SRL: 0449130, Mumbai, India) served as the positive control.

The medium was inoculated with MCC0226 to a final concentration of approximately 1 × 10^5^ CFU mL^−1^ and incubated at 37 °C under anaerobic conditions. Culture supernatants were collected after 24 and 48 h by centrifugation (5700× *g*, 10 min). The supernatants were acidified to pH 2.0 using 5 N HCl, centrifuged again to remove precipitates, and filtered through 0.22 μm membrane filters prior to chromatographic analysis.

SCFAs were quantified using a Shimadzu Ultra-Fast Liquid Chromatography (UFLC) system equipped with an Aminex HPX-87H column. Standard solutions of formic, acetic, propionic, butyric, valeric, lactic, caproic, and succinic acids were used for peak identification and quantification.

#### 2.4.7. Modulation of Tight Junction Protein (TJP) Expression

The effect of MCC0226 on the expression of intestinal tight junction genes was evaluated in the human colorectal epithelial cell line HT-29 (National Centre for Cell Science, Pune, India) following the method of Duary et al. [29] with minor modifications. HT-29 cells were cultured in Dulbecco’s Modified Eagle Medium (DMEM; Gibco, Waltham, MA, USA) and seeded into 24-well plates at a density of 1 × 10^5^ cells mL^−1^. Confluent cell monolayers were treated with MCC0226 at a final concentration of 1 × 10^8^ CFU mL^−1^ and incubated for 4 h at 37 °C. Untreated cells maintained in DMEM under identical conditions served as the control.

Following treatment, the monolayers were washed twice with phosphate-buffered saline (PBS), harvested by trypsinization, and total RNA was isolated using the RNeasy Mini Plus Kit (Qiagen, Germany). Complementary DNA (cDNA) was synthesized from purified RNA using the QuantiTect Reverse Transcription Kit (Qiagen, Germany). The relative expression of selected tight junction genes (Occludin (OCLN), Zona Occludin-1 (ZO-1), Claudin2 (CLDN-2), Claudin3 (CLDN-3) and Cadherin-1 (CDH-1) was quantified by reverse transcription quantitative PCR (RT-qPCR), and gene expression levels were normalized to the housekeeping gene GAPDH. Gene-specific primers used in this study are given in Table 1.

Relative expression of tight-junction proteins and CDH-1 was quantified by the comparative C_t_ (ΔΔC_t_) method using GAPDH as the endogenous reference. For each sample, ΔC_t_ was calculated as the difference between the mean C_t_ of the target TJP and the mean C_t_ of GAPDH, thereby normalizing target-gene expression to the housekeeping gene. ΔΔC_t_ was then obtained by subtracting the ΔC_t_ of the untreated control from the ΔC_t_ of the probiotic-treated sample, representing the treatment-induced change in normalized expression. Relative fold change was computed as 2^−ΔΔCt^, where values > 1 indicate upregulation and values < 1 indicate downregulation of TJP transcripts in treated cells relative to control.

### 2.5. Statistical Analysis

All experiments were conducted in triplicate, and the data are expressed as Mean ± SD. Data analysis was performed using the XLMiner Analysis Tool Pack. Statistical significance was determined using Student’s *t*-test or One-Way/Two-Way ANOVA followed by Tukey’s post-hoc test where appropriate. Differences were considered statistically significant at *p* < 0.05.

## 3. Results

### 3.1. Genomic Determinants Underlying the Probiotic Potential of MCC0226

Whole-genome sequencing of MCC0226 generated a draft genome of 2,047,110 bp with an average GC content of 32.9%. Genome-based taxonomic analysis confirmed MCC0226 as *L. salivarius*, with an average nucleotide identity (ANI) of 99.74% to the closest reference *L. salivarius* DSM 20555. Functional annotation using the RAST server identified 206 subsystems, comprising 3015 coding sequences (CDSs) and 89 RNA genes, indicating a broad functional repertoire associated with cellular metabolism, stress adaptation, and host interaction.

Genome annotation identified multiple genes associated with probiotic functionality (Table 2). Genes involved in acid and bile tolerance included the F_0_F_1_-ATPase complex, Na^+^/H^+^ antiporter, DnaK, DnaJ, GroEL, Clp proteases, HtrA, and cholylglycine hydrolase (BSH). In addition, several genes encoding adhesion-associated proteins were detected, including sortase A, mucus-binding proteins (MucBP/MBF), fibronectin-binding protein, and the surface-associated proteins enolase, EF-Tu, and GroEL.

The genome also contained a complete set of genes involved in exopolysaccharide biosynthesis, including epsC, epsD, and multiple glycosyltransferases responsible for EPS polymerization and assembly. Furthermore, genes associated with oxidative stress response and protein quality control, including NADH oxidase, peroxidase, thioredoxin/thioredoxin reductase, methionine sulfoxide reductases (MsrA/B), RecA, GroEL, GroES, Clp proteases, and HtrA, were identified.

**Figure 1:**
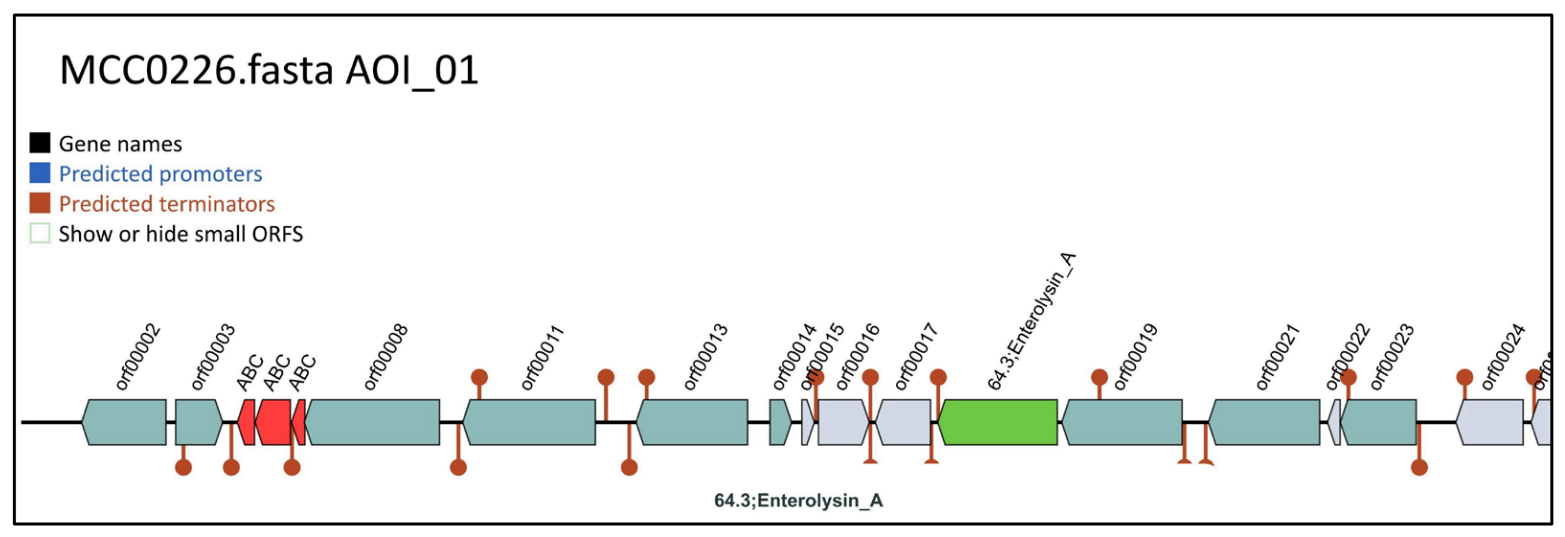
Enterolysin A Bacteriocin detected in MCC0226 by BAGEL4.0. (The genomic region was visualized using BAGEL. Gene names, predicted promoters, predicted terminators, and small ORFs are indicated according to the color coding of the BAGEL output).

Bacteriocin mining using BAGEL4 detected a gene cluster encoding Enterolysin A, a class III bacteriocin (Figure 1). No additional bacteriocin clusters were identified in the genome.

Analysis of metabolic pathways further identified complete biosynthetic pathways for the essential amino acids threonine and lysine (Table 3). Genes encoding aspartate-semialdehyde dehydrogenase, homoserine dehydrogenase, homoserine kinase, threonine synthase, diaminopimelate epimerase, diaminopimelate decarboxylase, aspartokinase, and other enzymes involved in lysine biosynthesis were present in the MCC0226 genome.

The genomic characterization of MCC0226 identified multiple genes associated with probiotic functionality and host interaction. Before evaluating these functional traits, the safety profile of MCC0226 was assessed through complementary genomic and phenotypic analyses to determine its suitability as a probiotic candidate.

### 3.2. Genomic and Phenotypic Safety Assessment

Genomic safety analysis using CARD, ResFinder, VirulenceFinder, PlasmidFinder, and PathogenFinder did not detect acquired virulence genes, toxin genes, plasmids, or transferable antimicrobial resistance determinants. CARD identified a putative vanT gene within the vanG cluster as a strict hit; however, the sequence showed less than 90% identity to reference antimicrobial resistance genes. Subsequent BLAST (BLAST+ 2.17.0) analysis indicated that this sequence represents an intrinsic chromosomal homolog rather than an acquired transferable resistance gene.

The antibiotic susceptibility profile of MCC0226 was determined using the E-test method, and the minimum inhibitory concentration (MIC) values were interpreted according to the EFSA microbiological cut-off values for Gram-positive bacteria (Table 4). MCC0226 was susceptible to ampicillin (0.75 μg mL^−1^), chloramphenicol (3 μg mL^−1^), streptomycin (32 μg mL^−1^), gentamicin (16 μg mL^−1^), erythromycin (0.75 μg mL^−1^), tetracycline (8 μg mL^−1^), and clindamycin (0.5 μg mL^−1^), with MIC values at or below the corresponding EFSA cut-off values. Gentamicin and tetracycline exhibited MIC values equal to the EFSA cut-off values and were therefore classified as susceptible. In contrast, MCC0226 exhibited an MIC > 256 μg mL^−1^ against kanamycin, exceeding the EFSA cut-off of 64 μg mL^−1^. Whole-genome analysis identified a putative GNAT-family acetyltransferase gene in the MCC0226 genome. BLAST analysis showed that this gene is homologous to GNAT-family acetyltransferases annotated in *L. salivarius* genomes.

Following confirmation of its safety profile, the ability of MCC0226 to survive gastrointestinal transit and establish interactions with the intestinal epithelium was investigated. Colonization-associated traits, including gastrointestinal tolerance, cell-surface properties, exopolysaccharide production, and epithelial adhesion, were evaluated to assess their potential for intestinal persistence.

### 3.3. MCC0226 Exhibits Traits Required for Intestinal Colonization

#### 3.3.1. Resistance to Gastrointestinal Conditions

The ability of MCC0226 to withstand simulated gastrointestinal conditions was evaluated using an initial inoculum of approximately 1 × 10^8^ CFU mL^−1^. MCC0226 remained highly viable during exposure to simulated gastric fluid (pH 2.5), with viable counts decreasing from 1.70 ± 0.38 × 10^8^ CFU mL^−1^ to 1.13 ± 0.03 × 10^8^ CFU mL^−1^ after 120 min of incubation (Table 5). This corresponded to a survival rate of 66.76%, with no detectable reduction in viable cell count on a logarithmic scale. Differences in viable counts across the gastric incubation period were statistically significant (paired *t*-test, *p* < 0.05).

The strain also exhibited sustained viability under simulated intestinal conditions containing bile salts and pancreatin. Following 360 min of exposure, MCC0226 retained 0.84 ± 0.20 × 10^8^ CFU mL^−1^, corresponding to 48.01% survival. Viable counts remained relatively stable during the initial stages of intestinal exposure, with survival values of 62.50% at 60 min, then gradually declining to 60.22% at 180 min and 48.01% after 360 min. Differences in viable counts across the intestinal incubation period were also statistically significant (paired *t*-test, *p* < 0.05).

#### 3.3.2. Surface Properties of MCC0226

**Figure 2:**
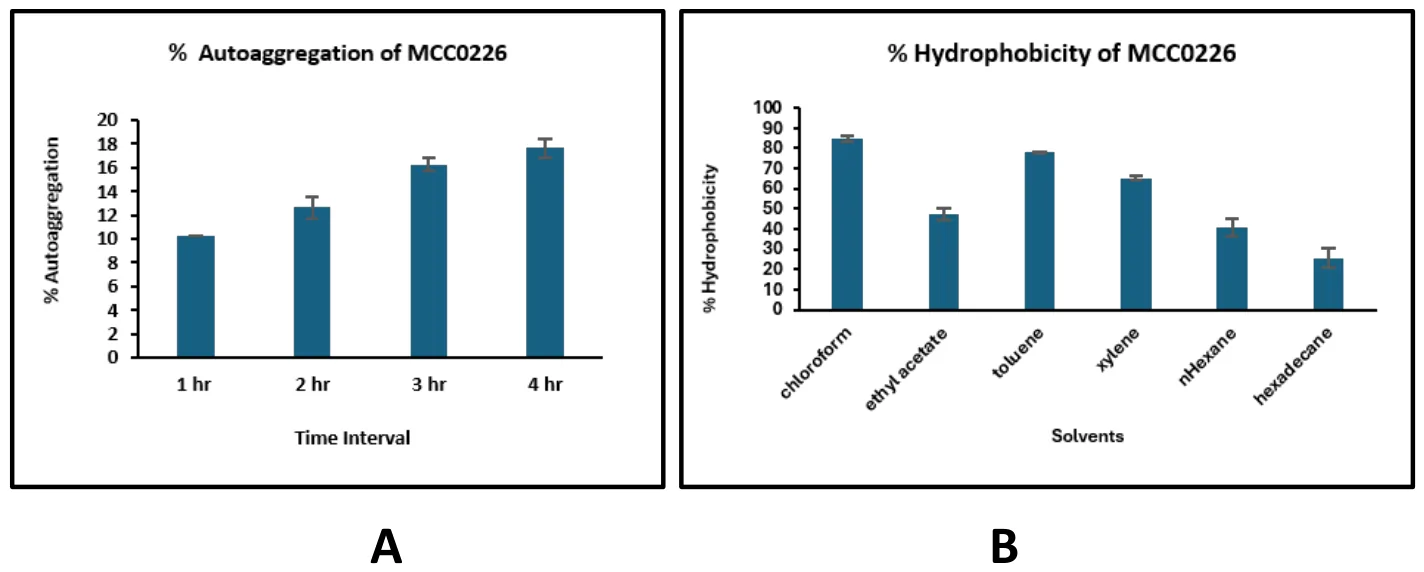
Adhesion-related surface properties of MCC0226: (**A**) autoaggregation and (**B**) cell surface hydrophobicity.

Surface-associated adhesion characteristics of MCC0226 were evaluated by autoaggregation and bacterial adhesion to hydrocarbons (BATH) assays (Figure 2A,B). MCC0226 exhibited a time-dependent increase in autoaggregation, reaching 17.65 ± 0.79% after 4 h of incubation. One-way ANOVA showed that the increase in autoaggregation over time was statistically significant (*p* < 0.05).

The BATH assay demonstrated high affinity of MCC0226 towards multiple hydrocarbons, with the highest hydrophobicity observed for chloroform (84.65 ± 1.24%), followed by toluene (77.98 ± 0.38%) and xylene (65.30 ± 1.09%). These results indicate a predominantly basic cell surface, which may facilitate interactions with the negatively charged intestinal mucosal surfaces. Differences in hydrophobicity among the hydrocarbons were statistically significant, as determined by one-way ANOVA (*p* < 0.05).

#### 3.3.3. Exopolysaccharide Production

MCC0226 produced measurable amounts of extracellular polysaccharides when grown under standard culture conditions. Quantification using the phenol–sulphuric acid assay showed a released extracellular EPS yield of 0.64 g L^−1^, confirming the strain’s ability to synthesize and secrete extracellular polysaccharides under the experimental conditions. The EPS quantification protocol used in this study measured soluble extracellular EPS recovered from the culture supernatant after protein removal and ethanol precipitation. Therefore, tightly cell-associated or capsular EPS was not efficiently recovered by this method, and the reported value may underestimate the total EPS produced by MCC0226.

#### 3.3.4. Adhesion to the Gut Epithelium

**Figure 3:**
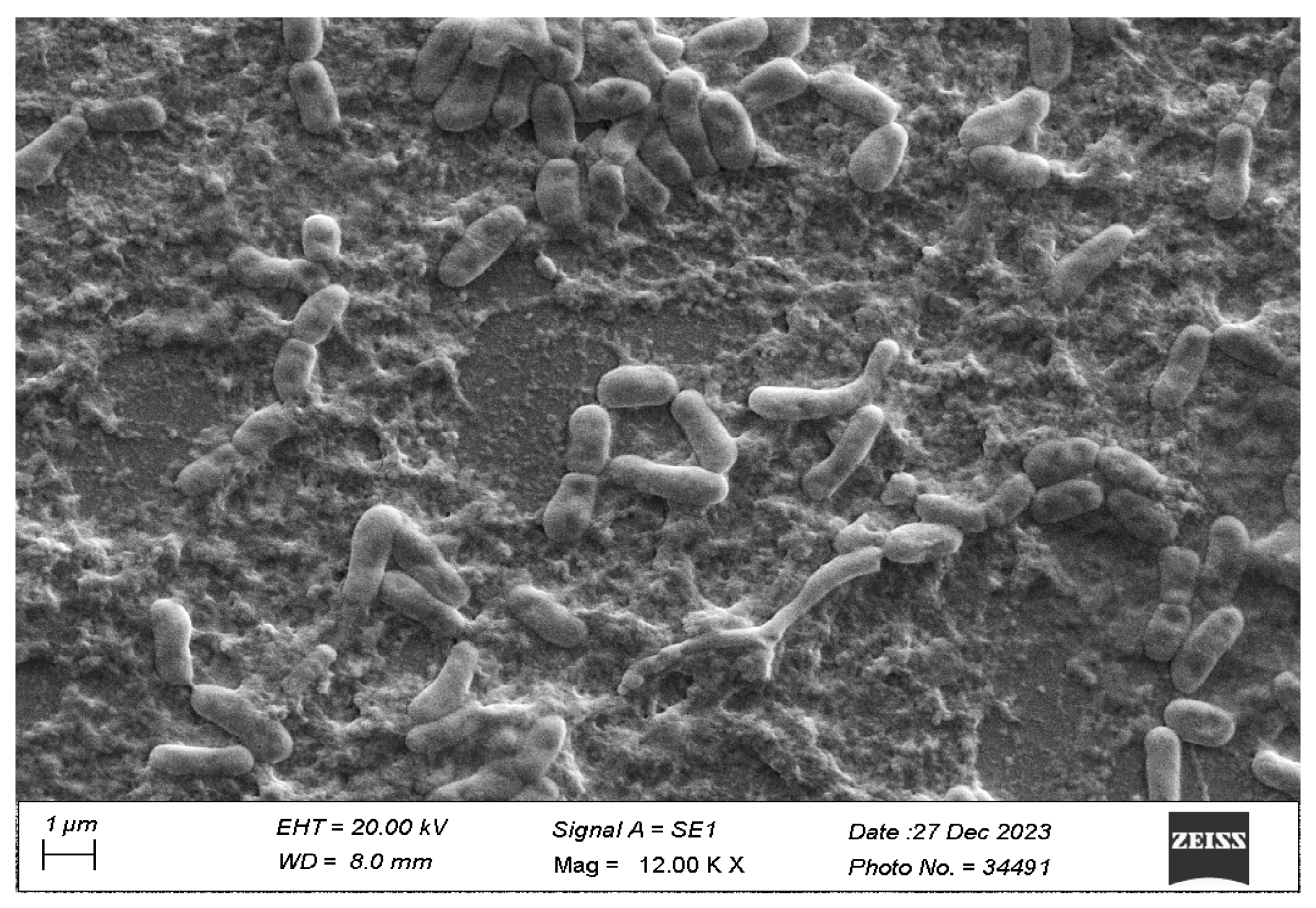
Scanning electron micrograph of adhered MCC0226 on Caco-2 cell line.

The adhesive ability of MCC0226 was further evaluated using HT-29 and Caco-2 intestinal epithelial cell lines. The strain adhered to both cell lines; however, a markedly higher adhesion index was observed for Caco-2 cells (944 ± 149 bacteria per 100 cells) than for HT-29 cells (153 ± 13.79 bacteria per 100 cells) (Figure 3). Scanning electron microscopy further confirmed the attachment of MCC0226 to the epithelial surface of Caco-2 cells, with bacterial cells observed in close association with the cell monolayer. The combined surface property assays and the epithelial adhesion assay demonstrate that MCC0226 has a strong capacity to attach to intestinal epithelial cells, supporting its potential for transient colonization of the intestinal mucosa.

Following the demonstration of intestinal colonization-associated traits, the ability of MCC0226 to interact with and inhibit enteric pathogens was examined using complementary assays evaluating antimicrobial activity, pathogen exclusion, coaggregation, and hydrogen peroxide production.

### 3.4. MCC0226 Modulates Microbial Interactions and Pathogen Exclusion

#### 3.4.1. Broad-Spectrum Antimicrobial Activity of MCC0226

**Figure 4:**
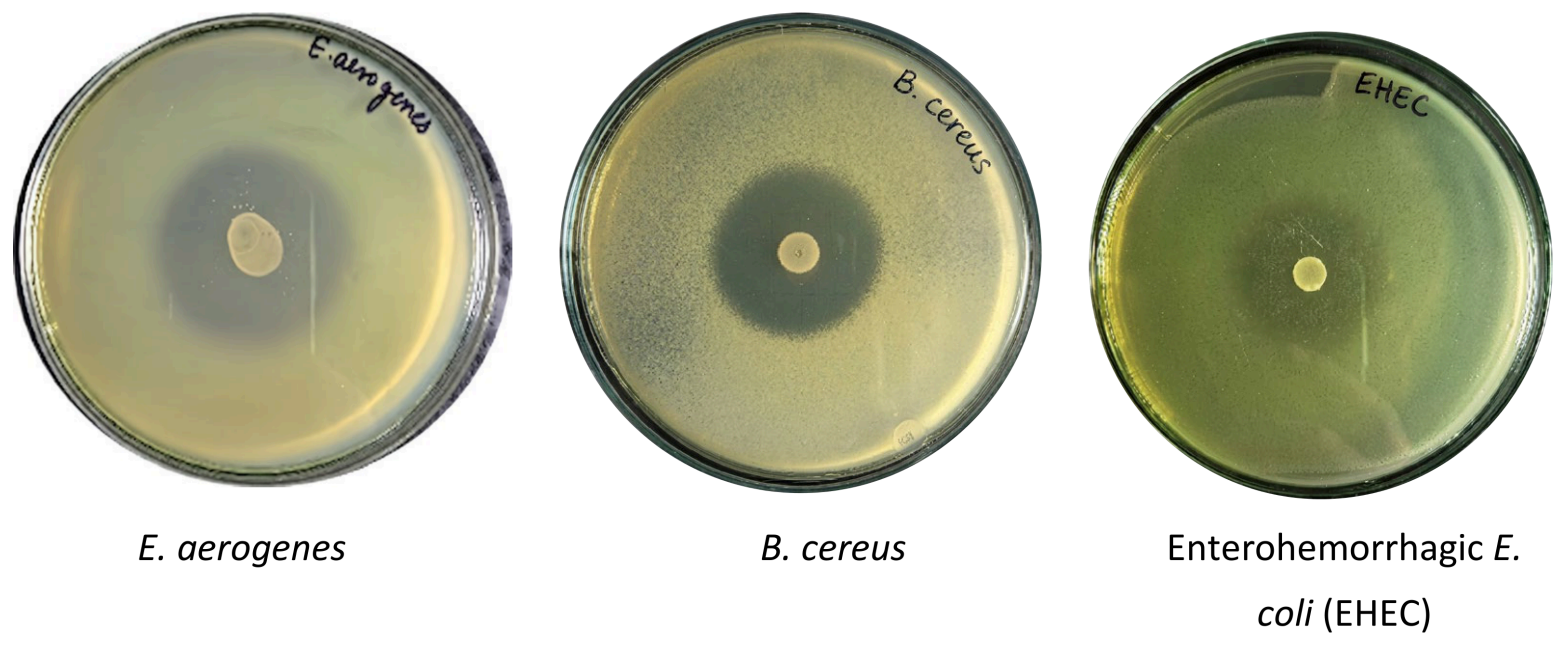
Agar spot assay demonstrating antimicrobial activity of MCC0226 against *E. aerogenes*, *B. cereus*, and EHEC.

The antimicrobial activity of MCC0226 was evaluated against a panel of fifteen clinically relevant bacterial pathogens using the agar spot assay (Table 6; Figure 4). MCC0226 inhibited 13 of the 15 pathogens tested, demonstrating a broad inhibitory spectrum against both Gram-negative and Gram-positive bacteria.

The largest inhibition zone was observed against *Y. enterocolitica* ATCC 23715 (40.5 ± 0.70 mm), followed by *S.* Typhimurium ATCC 13311 (37.5 mm) and *Enterobacter aerogenes* ATCC 13048 (36.5 ± 3.53 mm). Strong inhibition was also observed against *B. cereus* (31.66 ± 2.08 mm) and *K. pneumoniae* (25 ± 4.24 mm). Moderate inhibitory activity was detected against *E. coli* ATCC 8739 (19.0 ± 1.41 mm), enterohemorrhagic *E. coli* (EHEC) ATCC 43888 (23.3 ± 2.08 mm), *S. dysenteriae* (23.0 ± 2.82 mm), *E. faecalis* (15.0 ± 2.82 mm), *P. aeruginosa* (17.0 ± 0.0 mm), *C. perfringens* (15.33 ± 2.08 mm), and *S. aureus* (11.0 ± 1.41 mm). No inhibition was observed against *C. difficile* ATCC 9689 and *C. jejuni* ATCC 33560 under the conditions tested. The differences in antimicrobial activity among the tested pathogens were statistically significant (one-way ANOVA, *p* < 0.05).

#### 3.4.2. Antimicrobial Activity of the Cell-Free Supernatant

The antimicrobial activity of the cell-free supernatant (CFS) of MCC0226 was further evaluated using the agar well diffusion assay (Table 7). The 80% concentrated CFS inhibited all pathogens included in the assay, with inhibition zones ranging from 15.0 ± 1.0 mm to 24.0 ± 1.0 mm. The strongest activity was observed against *E. faecalis* (24.0 ± 1.0 mm), followed by *S.* Typhimurium (19.33 ± 2.88 mm), *P. aeruginosa* (19.0 ± 1.0 mm), *S. dysenteriae* (18.33 ± 1.52 mm), and *E. coli* ATCC 8739 (18.0 ± 3.46 mm).

Treatment of the CFS with proteinase K did not substantially alter the inhibition zones for the tested pathogens, indicating that antimicrobial activity was retained after proteolytic treatment. In contrast, neutralization of the CFS with NaOH abolished antimicrobial activity against all tested pathogens, indicating complete loss of inhibition following pH neutralization. The differences among the treatment groups were statistically significant (one-way ANOVA, *p* < 0.05). These findings demonstrate that the antimicrobial activity detected in the cell-free supernatant was primarily associated with acid-dependent metabolites under the tested culture conditions and do not exclude the possibility that bacteriocin production may occur under alternative physiological conditions.

#### 3.4.3. Pathogen Exclusion from Intestinal Epithelial Cells

The ability of MCC0226 to reduce pathogen adhesion to intestinal epithelial cells was evaluated using HT-29 monolayers. Pre-incubation of HT-29 cells with MCC0226 reduced the adhesion of both tested pathogens.

MCC0226 inhibited the adhesion of *S.* Typhimurium by 27.85 ± 4.40%, whereas a higher exclusion rate of 59.40 ± 13.65% was observed against *C. difficile*. The reduction in pathogen adhesion was greater for *C. difficile* than for *S.* Typhimurium under the experimental conditions.

#### 3.4.4. Coaggregation with Enteric Pathogens

**Figure 5:**
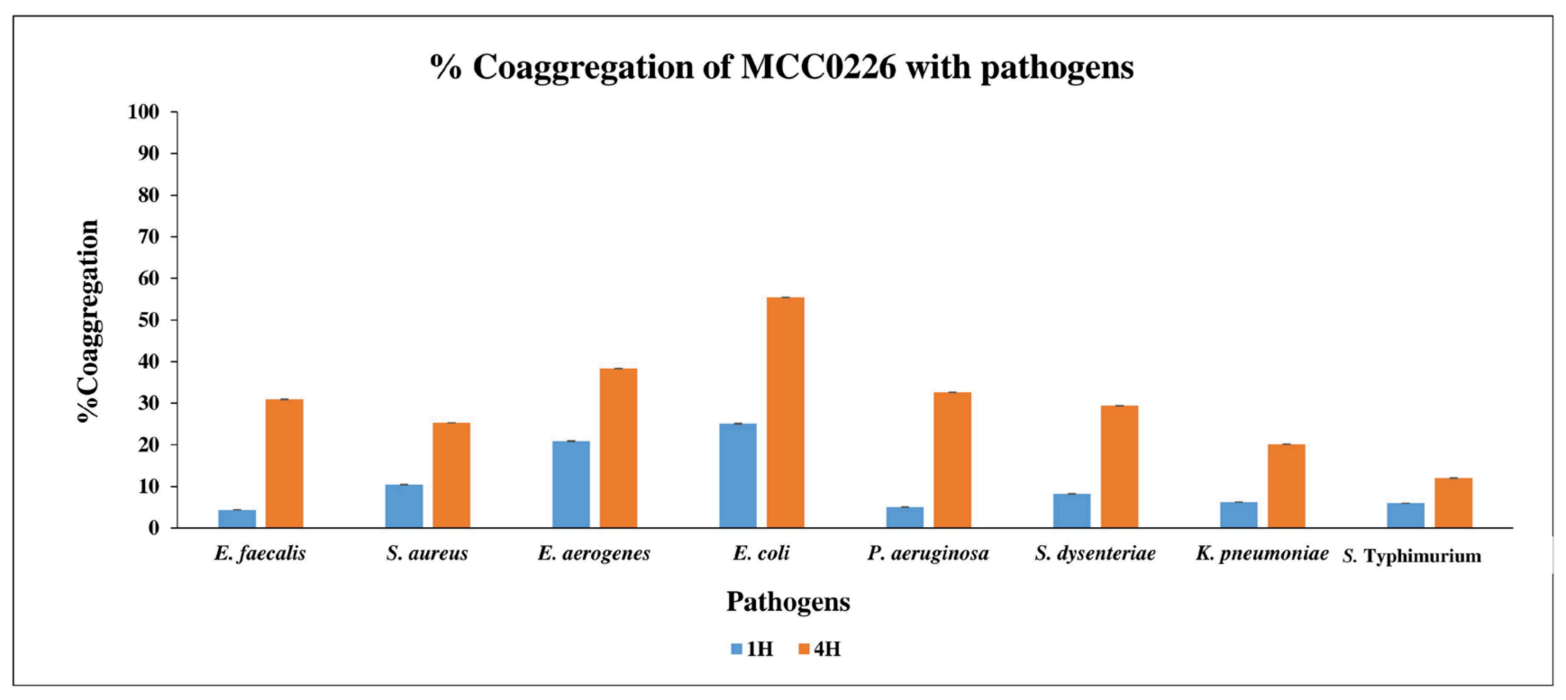
**%** Coaggregation of MCC0226 with pathogens.

The coaggregation ability of MCC0226 with pathogenic bacteria was assessed after 1 h and 4 h of incubation (Figure 5). Coaggregation increased with incubation time for all pathogens tested, indicating a time-dependent interaction between MCC0226 and the pathogens. The results demonstrated a significant, time-dependent increase in coaggregation (*p* < 0.05).

After 4 h, the highest coaggregation was observed with *E. coli* (55.47%), followed by *E. aerogenes* (38.34%). Lower coaggregation values were recorded with the remaining pathogens.

#### 3.4.5. Hydrogen Peroxide (H_2_O_2_) Production

Hydrogen peroxide production by MCC0226 was quantified after 24 h of incubation. The strain produced 0.56 ± 0.08 μg mL^−1^ of hydrogen peroxide under the assay conditions, confirming its ability to generate detectable levels of extracellular H_2_O_2_. This ability to generate H_2_O_2_ is consistent with one of the key mechanisms by which certain beneficial microorganisms inhibit competing microbes, thereby reinforcing the observed antimicrobial activity of MCC0226.

In addition to direct interactions with pathogenic microorganisms, probiotic-mediated gut homeostasis is influenced by the production of bioactive metabolites. Therefore, the metabolic functionality of MCC0226 was evaluated through antioxidant activity, cholesterol assimilation, bile salt hydrolase activity, and short-chain fatty acid production.

### 3.5. MCC0226 Produces Functional Metabolites Associated with Gut Barrier Homeostasis

#### 3.5.1. Antioxidant Activity

MCC0226 exhibited measurable antioxidant activity in the DPPH free radical scavenging assay, with a radical scavenging capacity of 41.40 ± 1.20%. These values demonstrate the strain’s capacity to neutralize free radicals, supporting its potential to mitigate oxidative stress.

#### 3.5.2. Anti-Hypercholesterolemic Activity

The cholesterol assimilation ability of MCC0226 was assessed under simulated intestinal (SIF) and simulated colonic (SCF) conditions. MCC0226 assimilated cholesterol in both simulated environments; however, significantly greater cholesterol assimilation was observed in SCF than in SIF. The strain removed 52.19 ± 0.71% of cholesterol from SCF, compared with 24.40 ± 0.65% from SIF.

Qualitative assessment of bile salt hydrolase (BSH) activity was performed on MRS agar supplemented with sodium glycocholate. MCC0226 produced distinct precipitation zones around the colonies, indicating positive BSH activity and the ability to deconjugate sodium glycocholate under the assay conditions.

#### 3.5.3. Short-Chain Fatty Acid Production (SCFA) Profile

The short-chain fatty acid (SCFA) production profile of MCC0226 was determined following growth in modified MRS medium supplemented with glucose, inulin, or fructooligosaccharides (FOS) as the carbon source. The metabolite profile varied depending on the carbohydrate substrate provided. Lactic acid was the predominant fermentation product under all conditions in which carbohydrate utilization occurred. The highest lactic acid concentration (4773.61 mg L^−1^) was detected in glucose-supplemented medium, while substantial production (2613.95 mg L^−1^) was also observed in the inulin-supplemented medium. Acetic acid was detected in both glucose- and inulin-containing media, whereas butyric acid reached 519.69 mg L^−1^ in the inulin-supplemented medium, representing the second most abundant metabolite under this condition. In contrast, MCC0226 showed no detectable utilization of fructooligosaccharides as a fermentation substrate, and no corresponding SCFA production was observed in FOS-supplemented medium. These results demonstrate substrate-dependent differences in the metabolic output of MCC0226, with glucose and inulin supporting fermentation and organic acid production under the experimental conditions. Genome-wide CAZyme annotation identified a GH32 family glycoside hydrolase, providing genomic evidence for the capacity of MCC0226 to metabolize inulin-type fructans.

Since microbial metabolites and probiotic–host interactions can influence epithelial barrier function, the effect of MCC0226 on intestinal tight junction gene expression was investigated in HT-29 cells. Expression of key junction-associated genes was quantified to assess the epithelial response to MCC0226 treatment.

### 3.6. MCC0226 Modulates Intestinal Epithelial Junction-Associated Gene Expression

**Figure 6:**
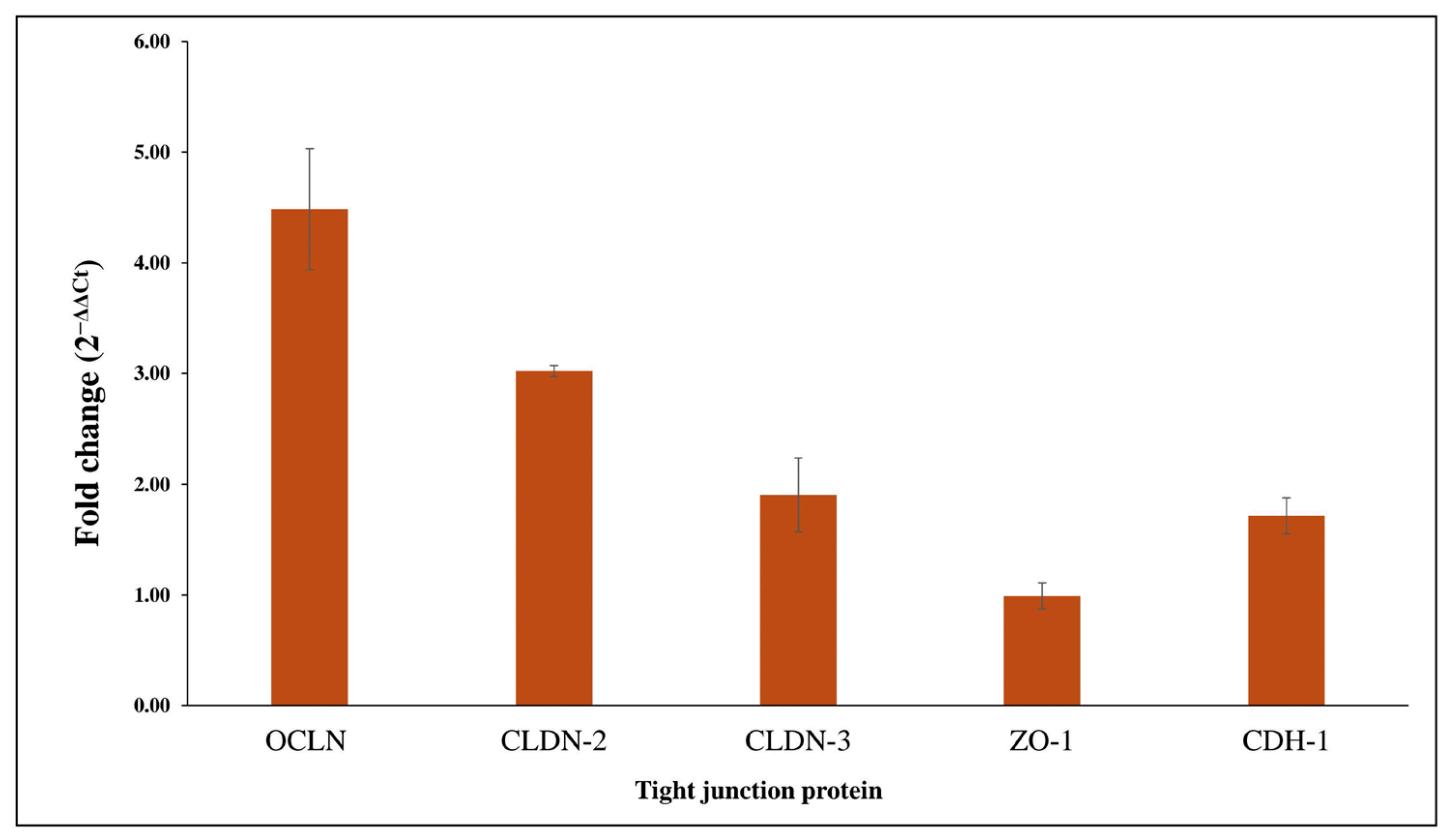
Effect of MCC0226 treatment on tight-junction gene expression in HT-29 cells. Fold changes were calculated using the 2^−ΔΔCt^ method relative to untreated control cells and normalized to the selected reference gene(s). Bars represent the mean ± SD of three technical replicates from one independent experiment.

The effect of MCC0226 on the expression of intestinal tight junction genes was evaluated in HT-29 cells following 4 h of treatment (Figure 6). RT-qPCR analysis showed differential expression of tight junction-associated genes in MCC0226-treated cells relative to untreated control. Among the genes analyzed, Occludin showed the greatest induction, with a 4.48-fold increase in expression. Claudin-2 was upregulated by 3.01-fold, followed by Claudin-3 (1.90-fold) and Cadherin-1 (1.71-fold). In contrast, the expression of ZO-1 remained unchanged, with a fold change of 0.99 compared with the control. The differences in gene expression between the control and MCC0226-treated cells were statistically significant, as determined by one-way ANOVA (*p* < 0.05).

These results indicate that MCC0226 modulated the transcriptional expression of multiple epithelial junction-associated genes in HT-29 cells under the experimental conditions. Since this analysis was based on RT-qPCR, the results represent changes in gene transcript abundance and do not directly assess epithelial barrier integrity or paracellular permeability.

## 4. Discussion

The present study provides an integrated genomic and functional characterization of *L. salivarius* MCC0226 and demonstrates that this human-derived strain possesses multiple complementary traits associated with gut ecosystem homeostasis and intestinal epithelial barrier protection. Rather than relying solely on conventional probiotic characterization, the study combined whole-genome sequencing with phenotypic assays to establish a mechanistic link between genomic determinants and functional probiotic attributes, including gastrointestinal survival, epithelial colonization, microbial interaction, metabolite production, and modulation of epithelial tight junction genes. This integrated approach aligns with the current understanding that probiotic efficacy is highly strain-specific and requires both genomic and functional validation before application in functional foods [4,32].

Whole-genome analysis established the molecular basis for the probiotic phenotype of MCC0226. The genome encoded genes involved in acid and bile tolerance (F_0_F_1_-ATPase, Na^+^/H^+^ antiporter, DnaK, DnaJ, GroEL, Clp proteases, HtrA, and bile salt hydrolase), adhesion (sortase A, mucus-binding proteins, fibronectin-binding proteins, enolase, and EF-Tu), exopolysaccharide biosynthesis, oxidative stress response, and bacteriocin production. Other probiotic *L. salivarius* strains from humans and animals carry similar genes, which support survival in the gut, interaction with epithelial cells, and antimicrobial activity [5,33]. Genome mining identified a biosynthetic gene cluster encoding Enterolysin A, a class III bacteriocin previously reported in *L. salivarius* and other lactic acid bacteria with lytic activity against several Gram-positive bacteria through peptidoglycan degradation [34]. The presence of this gene cluster demonstrates the genetic potential of MCC0226 to produce Enterolysin A; however, genomic identification alone does not establish its expression, secretion, or contribution to antimicrobial activity under the culture conditions examined in this study. In addition, genomic safety screening did not identify virulence factors, toxin genes, plasmids carrying resistance determinants, or clinically relevant transferable antimicrobial resistance genes. Although a vanT-like homolog was detected, its low sequence identity and chromosomal localization suggest it is likely an intrinsic genetic element rather than an acquired, transferable resistance determinant, a conclusion further supported by the phenotypic antibiotic susceptibility profile.

Safety is a prerequisite for probiotic applications in foods. Phenotypically, MCC0226 remained susceptible to seven of the eight antibiotics recommended by EFSA, including ampicillin, chloramphenicol, erythromycin, tetracycline, clindamycin, gentamicin, and streptomycin, while showing an elevated kanamycin MIC (>256 µg/mL), compared to the EFSA cut-off (64 µg/mL). Reduced susceptibility to aminoglycosides is frequently reported among lactobacilli and is generally attributed to limited intracellular uptake due to the low permeability of the bacterial cell membrane, rather than to acquired resistance mechanisms. Whole-genome analysis identified a putative GNAT-family acetyltransferase gene homologous to chromosomally encoded GNAT-family acetyltransferases reported in *L. salivarius* genomes. Although this gene represents a plausible intrinsic aminoglycoside resistance determinant, its contribution to the high kanamycin MIC observed in MCC0226 was not experimentally validated in the present study. The kanamycin-resistant phenotype may reflect intrinsic mechanisms characteristic of lactic acid bacteria, including limited aminoglycoside uptake associated with fermentative metabolism and other chromosomal resistance determinants, in addition to the predicted GNAT-family acetyltransferase.

This phenotypic profile is consistent with the genomic safety assessment and agrees with reports that intrinsic aminoglycoside resistance is common among several lactobacilli and is generally not associated with transferable resistance mechanisms [6]. The combined genomic and phenotypic evidence therefore supports the safety of MCC0226 for probiotic and functional food applications.

A defining characteristic of an effective probiotic is its ability to survive gastrointestinal transit and establish transient colonization of the intestinal mucosa. MCC0226 retained high viability under simulated gastric and intestinal conditions, maintaining 66.76% survival after 2 h of exposure to simulated gastric fluid and 48.01% survival after 6 h in simulated intestinal fluid without a substantial logarithmic reduction in viable counts. These observations are consistent with previous studies on *L. salivarius* strains L33 and F14, which also demonstrated high tolerance to acidic pH and bile salts [8,9]. The genomic identification of ATPase subunits, Na^+^/H^+^ antiporters, HtrA, and molecular chaperones provides a plausible genetic basis for this stress tolerance, suggesting that MCC0226 employs multiple mechanisms to maintain intracellular pH homeostasis and protein stability during gastrointestinal passage.

Colonization of the intestinal surface depends on both physicochemical surface properties and specific interactions with epithelial cells. MCC0226 exhibited moderate autoaggregation, along with high hydrophobicity towards chloroform, toluene, and xylene, indicating pronounced surface-interaction properties and a predominantly electron-donor (basic) cell surface. Cell-surface Hydrophobicity has been associated with improved interactions between probiotic bacteria and intestinal epithelial surfaces, whereas autoaggregation facilitates bacterial clustering and persistence within the intestinal environment [15]. Consistent with these surface characteristics, MCC0226 produced 0.64 g L^−1^ of exopolysaccharides, and its genome contained a complete EPS biosynthetic cluster including epsC, epsD, and glycosyltransferases. EPS production has previously been associated with enhanced adhesion, biofilm formation, and protection against environmental stress in *L. salivarius* and other lactobacilli [5,35].

Epithelial adhesion assays confirmed the functional relevance of these colonization-associated traits. MCC0226 adhered efficiently to both HT-29 and Caco-2 cells, with substantially greater adhesion to differentiated Caco-2 monolayers. This difference may reflect differences in the differentiation state and surface characteristics of the two intestinal epithelial cell models. Caco-2 cells undergo enterocyte-like differentiation and develop a brush-border phenotype, resulting in cellular and surface characteristics that may influence bacterial adhesion. However, the specific mechanisms underlying the differential adhesion observed in this study were not investigated. Scanning electron microscopy further confirmed the close association of bacterial cells with the epithelial surface. This phenotype is consistent with the presence of mucus-binding proteins, fibronectin-binding proteins, sortase-dependent LPXTG proteins, enolase, and EF-Tu identified in the genome. Similar adhesion-associated proteins have been reported to mediate *L. salivarius* interactions with mucins and extracellular matrix proteins, thereby promoting transient intestinal colonization and persistence within the gastrointestinal tract [5,10]. Together, these findings indicate that MCC0226 possesses complementary genomic and phenotypic characteristics required for successful colonization of the intestinal epithelium.

Following colonization, probiotics contribute to gut homeostasis by modulating interactions with resident and pathogenic microorganisms. MCC0226 exhibited broad-spectrum antimicrobial activity against 13 clinically relevant pathogens, including both Gram-negative enteric and Gram-positive gastrointestinal pathogens. Particularly strong inhibition was observed against *Y. enterocolitica*, *S.* Typhimurium, *E. aerogenes,* and *B. cereus*, indicating a broader inhibitory spectrum than that reported for many previously characterized *L. salivarius* strains, which have generally been evaluated against fewer indicator organisms [36]. The concentrated cell-free supernatant retained antimicrobial activity after proteinase K treatment but completely lost activity following pH neutralization, indicating that acidic metabolites were the predominant antimicrobial agents present in the tested supernatant under the experimental conditions. Organic acids, particularly lactic and acetic acids produced by lactobacilli, are well-established antimicrobial metabolites that inhibit enteric pathogens by lowering extracellular pH, disrupting membrane potential, and interfering with cellular metabolism [11]. Although Enterolysin A was identified through genome analysis, the absence of proteinase K sensitivity indicates that no detectable proteinaceous antimicrobial activity was present in the cell-free supernatant under the assay conditions used. Expression of Enterolysin A is known to be strain- and condition-dependent and may be influenced by growth phase, nutrient availability, environmental stress, quorum sensing, and pH. Since Enterolysin A transcription or protein production was not evaluated in this study, its expression under the experimental conditions remains to be determined.

MCC0226 also demonstrated additional mechanisms that may contribute to pathogen exclusion within the intestinal ecosystem. The strain reduced adhesion of both *S.* Typhimurium and *C. difficile* on HT-29 monolayers, with a greater exclusion effect observed against *C. difficile*. Pathogen exclusion is an important probiotic characteristic because occupation of epithelial binding sites limits pathogen attachment and subsequent colonization. In parallel, MCC0226 exhibited time-dependent coaggregation with several pathogens, reaching the highest coaggregation with *E. coli*. Coaggregation facilitates close physical association between probiotics and pathogens, thereby enhancing localized delivery of antimicrobial metabolites and reducing pathogen access to epithelial surfaces. Furthermore, MCC0226 produced detectable extracellular hydrogen peroxide, providing an additional antimicrobial mechanism commonly reported for beneficial lactic acid bacteria. Collectively, antimicrobial activity, pathogen exclusion, coaggregation, and hydrogen peroxide production indicate that MCC0226 modulates microbial interactions through multiple complementary strategies rather than a single antagonistic mechanism.

In addition to direct microbial interactions, probiotics contribute to gut homeostasis by producing metabolites that influence host physiology. MCC0226 exhibited appreciable antioxidant activity in the DPPH assay, demonstrating its capacity to scavenge free radicals in vitro. Oxidative stress is a major contributor to epithelial injury and intestinal inflammation, and antioxidant-producing lactobacilli have been proposed to mitigate oxidative damage in the gastrointestinal tract [5]. The experimentally observed antioxidant phenotype is consistent with the presence of oxidative stress response genes identified in the MCC0226 genome, including NADH oxidase, thioredoxin reductase, methionine sulfoxide reductases, and RecA.

MCC0226 also exhibited substantial cholesterol assimilation under simulated intestinal and colonic conditions, with significantly greater cholesterol removal in simulated colonic fluid. This activity was accompanied by positive bile salt hydrolase activity on sodium glycocholate-containing medium and by the presence of the cholylglycine hydrolase gene in the genome. Cholesterol reduction by probiotics is mediated through several complementary mechanisms, including bile salt deconjugation, cholesterol incorporation into bacterial membranes, and cholesterol precipitation during bacterial growth. The higher cholesterol assimilation observed under simulated colonic conditions suggests that MCC0226 remains metabolically active in environments representative of the large intestine, where fermentation predominates.

Fermentation of dietary substrates further demonstrated the metabolic versatility of MCC0226. Glucose and inulin supported the production of lactic acid as the predominant fermentation metabolite, together with acetic acid and measurable butyric acid in the inulin-containing medium. These metabolites contribute to intestinal homeostasis, with butyrate serving as an important energy source for colonocytes and supporting epithelial physiology, while acetate and lactate contribute to maintenance of the intestinal microbial environment [37,38]. Interestingly, MCC0226 did not exhibit detectable growth or short-chain fatty acid production when fructooligosaccharides (FOS) were provided as the sole carbon source, indicating substrate-specific fructan utilization under the experimental conditions. To investigate the genomic basis of this phenotype, CAZyme annotation using dbCAN identified a GH32 family glycoside hydrolase in the MCC0226 genome (contig_1_632). GH32 enzymes include β-fructofuranosidases, exo-inulinases, and fructan β-fructosidases that hydrolyze β-(2→1)-linked fructans, including inulin and fructooligosaccharides, and are key enzymes involved in microbial fructan metabolism. The presence of GH32 is therefore consistent with the observed utilization of inulin by MCC0226. However, the inability to utilize the FOS preparation used in this study suggests that fructan metabolism is influenced by factors beyond the presence of GH32 alone, including substrate degree of polymerization, transporter specificity, and regulation of carbohydrate metabolism. Similar substrate-specific utilization of fructans has been reported in probiotic bacteria, where distinct fructan transport systems and CAZyme repertoires determine preferential utilization of inulin or specific FOS molecules [39]. Therefore, inulin appears to be a more suitable prebiotic substrate than the FOS preparation tested here for future synbiotic formulations involving MCC0226, although the molecular basis of this substrate preference requires further investigation.

One of the important findings of this study is that MCC0226 modulated the transcriptional expression of several epithelial junction-associated genes. Treatment with MCC0226 increased the transcript levels of OCLN, CLDN3, CDH1,, and CLDN2, while ZO-1 levels remained unchanged. CLDN2, a pore-forming claudin, mediates paracellular cation/water flux via the distinct “pore pathway,” which is mechanistically separable from the “leak” and “unrestricted” pathways governed by occludin, CLDN3, and other barrier-forming proteins [40,41]. Its co-induction with sealing junctional genes need not indicate leakiness: CLDN2 upregulation can reflect adaptive physiological responses (e.g., fluid secretion, pathogen clearance) [41], be counteracted post-translationally via autophagic degradation of membrane-bound CLDN2 [42] or occur alongside dominant barrier-strengthening effects from CLDN3/OCLN/CDH1, yielding net-protective phenotypes despite CLDN2’s individual permeabilizing function [42]. Thus, the concurrent induction of CLDN2 with OCLN, CLDN3 and CDH1 represents a mixed transcriptional response rather than conclusive evidence of either increased epithelial leakiness or improved barrier function. The functional significance of CLDN2 expression is also dependent on its abundance, cellular localization, and post-transcriptional regulation. Because the present study evaluated transcript expression only and did not assess TEER, paracellular permeability, or junctional protein abundance and localization, the net effect of MCC0226 on epithelial barrier function cannot be determined. Further functional studies are therefore required to establish whether the observed transcriptional changes translate into altered epithelial permeability.

Previous studies have reported that *L. salivarius* strains can modulate epithelial junction-associated proteins and influence epithelial barrier function in intestinal models under both basal and inflammatory conditions. *L. salivarius* CNCM I-4866 has been shown to improve epithelial barrier function by modulating tight junction-associated responses [10], while other *L. salivarius* strains have been reported to regulate the expression of Occludin, Claudins, ZO-1, and mucins in intestinal epithelial cells [5]. However, unlike those studies, the present work did not include functional barrier assays such as transepithelial electrical resistance (TEER), FITC-dextran or mannitol permeability assays, or immunolocalization of junctional proteins. Therefore, whether MCC0226 ultimately exerts a barrier-protective, neutral, or context-dependent effect on epithelial integrity requires further investigation using complementary functional assays.

The multifunctional phenotype of MCC0226 suggests several mechanisms through which the strain may benefit the host, including persistence within the gastrointestinal tract, competitive exclusion of enteric pathogens, production of beneficial metabolites, cholesterol metabolism, and modulation of epithelial junction-associated gene expression. These complementary traits support the potential application of MCC0226 in functional foods and synbiotic formulations aimed at promoting intestinal microbial homeostasis. However, functional studies evaluating epithelial barrier integrity and host immune responses are required to confirm these potential health benefits.

## 5. Conclusions

This study provides a comprehensive genomic and functional characterization of *L. salivarius* MCC0226 as a putative probiotic candidate with multiple traits associated with probiotic potential. MCC0226 exhibited a favorable safety profile, survived simulated gastrointestinal conditions, adhered to intestinal epithelial cells, and demonstrated diverse functional attributes, including antimicrobial activity, exopolysaccharide production, antioxidant activity, bile salt hydrolase activity, cholesterol assimilation, pathogen exclusion, and substrate-specific carbohydrate fermentation. The strain selectively utilized inulin, but not the fructooligosaccharide preparation evaluated in this study, for growth and short-chain fatty acid production, highlighting a substrate preference that may be relevant for the development of strain-specific synbiotic formulations. MCC0226 also modulated the transcriptional expression of epithelial junction-associated genes (OCLN, CLDN3, CDH1, and CLDN2) in HT-29 cells, indicating its ability to influence host epithelial responses under the experimental conditions. Overall, the combined genomic and in vitro findings identify MCC0226 as a promising candidate for further investigation in functional food and nutraceutical applications. However, the present observations are based on genomic, in vitro, and cell-based assays, and future studies employing functional barrier assays, advanced intestinal models, and in vivo validation are required to establish the physiological relevance and clinical efficacy of MCC0226 and to elucidate the contributions of specific metabolites and bacteriocins to its host-associated effects.

## Figures and Tables

**Table 1:** Primer sequences of Tight Junction genes used for RT-qPCR analysis.

Gene	Gene ID	Primer Sequence	Reference
**GAPDH**	NM_002046.7	F: TGACTTCAACAGCGACACCCA R: CACCCTGTTGCTGTAGCCAAA	[30]
**OCLN**	NM_002538.4	F: ACCAATGCTCTCTCAGCCAG R: AGGCAAAGATGGCAATGCAC	
**CDH-1**	NM_004360.5	F: CTTTGACGCCGAGAGCTACA R: TTTGAATCGGGTGTCGAGGG	
**CLDN-2**	NM_020384.4	F: TATAGCACCCTTCTGGGCCT R: GCTACCGCCACTCTGTCTTT	
**CLDN-3**	NM_001306	F: CTACGACCGCAAGGACTACG R: GTGGTGGTGTTGGTGGTG	[31]
**ZO-1**	NM_001301025.3	F: GTGCTGGCTTGGTCTGTTTG R: TCTGTACATGCTGGCCAAGG	Primers were designed in silico in the present study.

**Table 2:** Genes encoding for probiotic attributes of MCC0226.

Category	Key Genes/Proteins	Predicted Function
**Acid and bile tolerance**	ATP synthase (F_0_F_1_-ATPase subunits); Na^+^/H^+^ antiporter	Acid tolerance via pH homeostasis
	DnaK, DnaJ, GroEL, Clp proteases (ClpC/X/P)	Protection & repair of proteins under acid stress
	Cholylglycine hydrolase (BSH); Sortase A; HtrA	Bile tolerance
**Adhesion and colonization**	Sortase A (LPXTG-specific); Mucus-binding factor (MBF/MucBP)	Binding to mucin & extracellular matrix; adhesion
	Fibronectin-binding protein	Binding to fibronectin; auto-/co-aggregation
	Enolase; EF-Tu; GroEL	Moonlighting surface proteins mediating host binding
**Exopolysaccharide (EPS) biosynthesis**	EpsC (Wze); EpsD (Wzd); glycosyltransferases (e.g., EpsE, EpsF) Tyrosine-protein kinase/phosphatase regulators	Polymerization, chain-length determination, and repeating-unit synthesis Regulatory control of EPS production
**Oxidative stress response & protein quality control**	NADH oxidase/peroxidase; Thioredoxin system (Trx/TrxR); MsrA/B Rec A GroEL, GroES, Clp proteases, HtrA	Detoxification of O_2_/H_2_O_2_ & redox homeostasis DNA repair Targeting, degradation, and proper folding of misfolded proteins

**Table 3:** Amino acid biosynthesis, protein/gene/system detected in the MCC0226 genome.

Description	Amino Acid Biosynthesis, Protein/Gene/System Detected in the Genome
Threonine	Aspartate-semialdehyde dehydrogenase homoserine dehydrogenase homoserine kinase threonine synthase
Lysine	Diaminopimelate epimerase (EC 5.1.1.7) Diaminopimelate decarboxylase (EC 4.1.1.20) Aspartokinase (EC 2.7.2.4) Aspartate-semialdehyde dehydrogenase (EC 1.2.1.11) 4-hydroxy-tetrahydrodipicolinate synthase (EC 4.3.3.7) 4-hydroxy-tetrahydrodipicolinate reductase (EC 1.17.1.8) 2,3,4,5-tetrahydropyridine-2,6-dicarboxylate N-acetyltransferase (EC 2.3.1.89)

**Table 4:** Antibiotic susceptibility profile of MCC0226.

Sr. No	Antibiotics	MIC Value	EFSA MIC Cut-Off	Interpretation
**1**	Ampicillin	0.75	4	S
**2**	Chloramphenicol	3	4	S
**3**	Streptomycin	32	64	S
**4**	Gentamicin	16	16	S
**5**	Kanamycin	>256	64	R
**6**	Erythromycin	0.75	1	S
**7**	Tetracycline	8	8	S
**8**	Clindamycin	0.5	4	S

**Table 5:** Survival of MCC0226 in simulated gastrointestinal conditions at different time intervals.

Condition	Time Interval (min)	Total Viable Count (CFU/mL)	Survival (%)
**Simulated gastric fluid**	0	1.70 ± 0.38 × 10^8^	100
	60	1.35 ± 0.25 × 10^8^	79.41
	120	1.13 ± 0.03 × 10^8^	66.76
**Simulated intestinal fluid**	0	1.76 ± 0.47 × 10^8^	100
	60	1.1 ± 0.21 × 10^8^	62.50
	180	1.06 ± 0.21 × 10^8^	60.22
	360	0.84 ± 0.20 × 10^8^	48.01

**Table 6:** Antimicrobial activity of MCC0226 against pathogens using agar spot assay.

Sr. No	Pathogens	Zone of Inhibition (mm) Observed Against Pathogens by MCC0226
1	*E. coli* ATCC 8739	19 ± 1.41
2	*S. aureus* ATCC 6538	11 ± 1.41
3	*S. dysenteriae* ATCC 13313	23 ± 2.82
4	*E. aerogenes* ATCC 13048	36.5 ± 3.53
5	*K. pneumoniae* ATCC 13883	25 ± 4.24
6	*E. faecalis* ATCC 29212	15 ± 2.82
7	*P. aeruginosa* ATCC 10145	17 ± 0
8	*S.* Typhimurium ATCC 13311	37.5 ± 0.70
9	*B. subtilis* ATCC 6051	9 ± 0
10	*C. perfringens* ATCC 13124	15.33 ± 2.08
11	*E. coli* (EHEC) ATCC 43888	23.3 ± 2.08
12	*B. cereus* ATCC 10876	31.66 ± 2.08
13	*Y. enterocolitica* ATCC 23715	40.5 ± 0.70
14	*C. difficile* ATCC 9689	No zone
15	*C. jejuni* ATCC 33560	No zone

**Table 7:** Inhibitory activity of cell-free supernatant (CFS) of MCC0226 against pathogens.

Sr. No	Pathogens	Zone of Inhibition (mm) Observed Against Pathogens by MCC0226	
		80% Conc. CFS	80% Conc. CFS Treated with Proteinase K
1	*E. coli* ATCC 8739	18 ± 3.46	16 ± 3.46
2	*S. aureus* ATCC 6538	16.33 ± 0.57	17 ± 1
3	*S. dysenteriae* ATCC 13313	18.33 ± 1.52	17 ± 2.64
4	*E. aerogenes* ATCC 13048	16 ± 0	17.5 ± 2.12
5	*K. pneumoniae* ATCC 13883	15 ± 1	15 ± 2
6	*E. faecalis* ATCC 29212	24 ± 1	22.33 ± 2.08
7	*P. aeruginosa* ATCC 10145	19 ± 1	19.33 ± 1.52
8	*S.* Typhimurium ATCC 13311	19.33 ± 2.88	19.33 ± 2.08
9	*B. subtilis* ATCC 6051	17 ± 1	18 ± 0

## Data Availability

The MCC0226 genome has been deposited at NCBI GenBank under the nucleotide sequence accession number JBANDJ000000000.1.
